# Inhibition of Soluble Epoxide Hydrolase Limits Mitochondrial Damage and Preserves Function Following Ischemic Injury

**DOI:** 10.3389/fphar.2016.00133

**Published:** 2016-06-07

**Authors:** Maria K. Akhnokh, Feng Hua Yang, Victor Samokhvalov, Kristi L. Jamieson, Woo Jung Cho, Cory Wagg, Abhijit Takawale, Xiuhua Wang, Gary D. Lopaschuk, Bruce D. Hammock, Zamaneh Kassiri, John M. Seubert

**Affiliations:** ^1^Faculty of Pharmacy and Pharmaceutical Sciences, 2-020M Katz Group Centre for Pharmacy and Health Research, University of AlbertaEdmonton, AB, Canada; ^2^Guangdong Laboratory Animal Monitoring InstituteGuangdong, China; ^3^Imaging Core Facility, Faculty of Medicine and Dentistry, University of AlbertaEdmonton, AB, Canada; ^4^Mazankowski Alberta Heart Institute, University of AlbertaEdmonton, AB, Canada; ^5^Department of Pharmacology, Faculty of Medicine and Dentistry, University of AlbertaEdmonton, AB, Canada; ^6^Department of Physiology, Faculty of Medicine and Dentistry, University of AlbertaEdmonton, AB, Canada; ^7^Department of Pediatrics, Faculty of Medicine and Dentistry, University of AlbertaEdmonton, AB, Canada; ^8^Department of Entomology and Nematology Comprehensive Cancer Center, University of California, DavisDavis, CA, USA

**Keywords:** acute myocardial infarction, mitochondrial efficiency, soluble epoxide hydrolase, arachidonic acid

## Abstract

**Aims:** Myocardial ischemia can result in marked mitochondrial damage leading to cardiac dysfunction, as such identifying novel mechanisms to limit mitochondrial injury is important. This study investigated the hypothesis that inhibiting soluble epoxide hydrolase (sEH), responsible for converting epoxyeicosatrienoic acids to dihydroxyeicosatrienoic acids protects mitochondrial from injury caused by myocardial infarction.

**Methods:** sEH null and WT littermate mice were subjected to surgical occlusion of the left anterior descending (LAD) artery or sham operation. A parallel group of WT mice received an sEH inhibitor, trans-4-[4-(3-adamantan-1-y1-ureido)-cyclohexyloxy]-benzoic acid (tAUCB; 10 mg/L) or vehicle in the drinking water 4 days prior and 7 days post-MI. Cardiac function was assessed by echocardiography prior- and 7-days post-surgery. Heart tissues were dissected into infarct, peri-, and non-infarct regions to assess ultrastructure by electron microscopy. Complexes I, II, IV, citrate synthase, PI3K activities, and mitochondrial respiration were assessed in non-infarct regions. Isolated working hearts were used to measure the rates of glucose and palmitate oxidation.

**Results:** Echocardiography revealed that tAUCB treatment or sEH deficiency significantly improved systolic and diastolic function post-MI compared to controls. Reduced infarct expansion and less adverse cardiac remodeling were observed in tAUCB-treated and sEH null groups. EM data demonstrated mitochondrial ultrastructure damage occurred in infarct and peri-infarct regions but not in non-infarct regions. Inhibition of sEH resulted in significant improvements in mitochondrial respiration, ATP content, mitochondrial enzymatic activities and restored insulin sensitivity and PI3K activity.

**Conclusion:** Inhibition or genetic deletion of sEH protects against long-term ischemia by preserving cardiac function and maintaining mitochondrial efficiency.

## Introduction

Arachidonic acid (AA) is a polyunsaturated fatty acid found in the phospholipid domain of cell membranes. Activation of cytoplasmic phospholipase A2 and other enzymes trigger the release of AA, which is further metabolized into a vast array of lipid mediators. The predominant enzyme systems that metabolize AA are cyclooxygenase, lipoxygenase, and cytochrome P450 (CYP) monooxygenases generating prostanoids, leukotrienes, epoxidized, and hydrolylated metabolites, respectively (Wang and Dubois, 2012). CYP epoxygenases such as CYP2J and CYP2C isozymes metabolize AA into biologically active lipid mediators, called epoxyeicosatrienoic acids (5,6-EET, 8,9-EET, 11,12-EET, 14,15-EET), which have important roles in the cardiovascular system (Spector and Kim, 2015). While removal of EETs may occur by conjugation, chain elongation, β-oxidation, and esterification into phospholipid membranes, the predominant pathway is metabolism of EETs into the less active vicinal diol compounds by soluble epoxide hydrolase (sEH) (Spector and Kim, 2015). An approach to increase cellular EET levels and overcome their rapid metabolism is to inhibit sEH activity (Morisseau and Hammock, 2013). Inhibition of sEH has been associated with decreased atherosclerotic plaque lesions in mice aortae (Ulu et al., 2008), decreased blood pressure in hypertensive mice (Neckář et al., 2012) and protection against ischemic injury (Seubert et al., 2006; Li et al., 2009; Batchu et al., 2012a). Other effects of inhibiting sEH include vasodilation, pro-angiogenisis, and cell migratory effects (Imig and Hammock, 2009; Oni-Orisan et al., 2014).

In the heart, mitochondria provide the primary source of energy to fuel the contractile machinery. The heart's high-energy demand during normal function is met by a continuous supply of ATP mainly produced through oxidative phosphorylation in mitochondria. Mitochondria are strategic regulators of cell life and death given the fact that they play a central role in energy production, calcium homeostasis, and stress adaptation (Jendrach et al., 2008). These dynamic organelles undergo continuous fusion and fission processes in response to cellular energy demands and stress levels. As cardiomyocytes are terminally differentiated post-mitotic cells, maintenance of a healthy pool of mitochondria depends upon a delicate balance between newly generated organelles and efficient degradation of irreversibly damaged organelles (Hom and Sheu, 2009). During ischemic stress, several signaling pathways affect mitochondrial function and structure, which can impact ionic gradients and initiate cell death pathways. These changes lead to uncoupling of electron flow, opening of the mPTP and loss of cytochrome c, leading to mitochondrial dysfunction and eventually irreversible cell death.

We have previously demonstrated that EETs enhance cardiomyocyte cell survival via a protective cascade targeting the mitochondria (Katragadda et al., 2009; Batchu et al., 2012a; Samokhvalov et al., 2013, 2014). Emerging evidence suggests the cardioprotective effect of EETs is due to inhibition of mitochondrial damage. For instance, EETs limit mitochondrial damage and fragmentation following IR injury in CYP2J2 overexpressing mice compared to wild type littermates (Katragadda et al., 2009). In addition, EETs minimize doxorubicin-induced mitochondrial dysfunction, and damage preventing cardiotoxicity (Zhang et al., 2009). sEH inhibition has been shown to maintain mitochondrial membrane potential (ΔΨ_m_) following cellular stress limiting mitochondrial dysfunction (Batchu et al., 2012a). The present study investigates the effect of sEH inhibition in maintenance of mitochondrial efficiency following myocardial infarction.

## Methods

### Animals

A colony of mice on a C57/BL6 background with targeted disruption of the sEH gene (*EPHX*) and wild-type (WT) littermates are maintained at the University of Alberta (Seubert et al., 2006). All studies were carried out using 2–3 month old mice weighing 25–30 g. To pharmacologically inhibit sEH,10 mg/L tAUCB was administered to WT mice in drinking water 4 days prior to surgery and continued for 7 days after surgery (Hwang et al., 2007). Vehicle (DMSO; 1 μl/ml) was added to the drinking water of the sEH null and WT littermates. Mice were euthanized with an intra-peritoneal injection of sodium pentobarbital (100 mg/kg) and checked to ensure the absence of movement, flexor, and pedal reflexes prior to tissue collection. Experiments were conducted according to strict guidelines of the Canadian Council on Animal Care, Use of Laboratory Animals published by the US National Institute of Health (NIH publication, 8th edition, 2011) and were approved the University of Alberta Health Sciences Animal Welfare Committee.

### Myocardial infarction (MI)

MI was induced by permanent occlusion of the proximal left anterior descending (LAD) coronary artery as described (Kandalam et al., 2010). Mice were anesthetized with ketamine (100 mg/kg) and xylazine (10 mg/kg), intubated, and underwent left thoracotomy in which LV was exposed by opening the pericardium and the LAD was encircled and ligated. The mortality rate following surgery was < 2% in all groups. In sham-operated mice, the LAD was encircled but not ligated. On day 7 post-MI, mice were euthanized and hearts were collected from sham-operated and post-MI mice, and LV was dissected into infarct, peri-, and non-infarct regions using a dissecting microscope. We first identified the suture node 2–3 mm under apex of the left atrium. A pale (gray) area from the node toward the apex of the heart could be visualized; this region was identified as infarction. Tissue with good blood supply and normal wall thickness were identified non-infarct area. A 2 mm narrow line along the each side of the infarct region was set as peri-infarct region. The different regions were separately flash-frozen using liquid nitrogen and stored at −80°C for further analysis.

### Infarct size analysis

Hearts were sliced from apex to the point of ligation in 0.5-mm slices. Slices were then incubated in 1% triphenyltetrazolium chloride at 37°C for 10 min. In viable tissues, TTC is reduced by dehydrogenases to 1,2,5-triphenylformazan, which has a brick red color. In necrotic tissues, TTC will remain white due to the absence of enzymes. The percentage of infarct was determined from cross-sections of the whole left ventricle, which was set as 100%. Images were analyzed using ImageJ software (Kandalam et al., 2010).

### sEH activity

Non-infarct regions of LV from post-MI and sham hearts were flash frozen in liquid nitrogen immediately after harvesting. Frozen tissues were crushed using mortar and pestle. Heart powders were homogenized in ice-cold homogenization buffer (250 mM sucrose, 10 mM Tris-HCl, and phosphatase protease inhibitor). sEH activity was assessed using a soluble epoxide hydrolase assay kit assay (Cat # 600090, Cayman Chemical).

### Echocardiography measurements

Non-invasive functional assessment was performed by transthoracic echocardiography using a Vevo 770 high-resolution imaging system with a 30 MHz transducer (RMV-707B; VisualsSonics). Isoflurane (0.8% by anesthetic machine) was used to anesthetize the mice during the recordings. To assess changes in cardiac function, echocardiography was carried 1 day prior to MI (baseline) and 7 days post-MI. Left ventricular end-systolic diameter (LVESD) and end-diastolic diameter (LVEDD) were obtained from M-mode images, while left atrial size was obtained by M-mode imaging in the parasternal long axis view (Wang et al., 2013). Systolic function was assessed by calculating ejection fraction (%EF) and fractional shortening (%FS) using the following equations %EF = [(LVEDV − LVESV)/LVEDV] × 100 and %FS = [(LVEDD − LVESD)/LVEDD] × 100. Tei index was calculated as (isovolumic contraction time (IVCT) + isovolumic relaxation time (IVRT))/ejection time (ET). Diastolic function was represented as early transmitral LV filling wave (E-wave) and late LV filling wave (A-wave), which were measured using pulsed-wave Doppler tissue imaging as described in (Basu et al., 2009; Kandalam et al., 2010). VisualSonics software was used for the qualitative and quantitative measurements.

### Mitochondrial ultrastructure

Mitochondrial ultrastructure was assessed in several pieces obtained from different regions of the left ventricle that were pre-fixed in 2.5% glutaraldehyde in 0.1M sodium cacodylate buffer, post-fixed in 2% osmium tetroxide (OsO4) in 0.1M sodium cacodylate buffer, dehydrated in an ethyl alcohol series, embedded with epoxy resin, and thermally polymerized as previously described (Katragadda et al., 2009). Ultra thin-sections (60 nm) were cut by an ultramicrotome (Leica UC7, Leica Microsystems Inc., ON, Canada) and then stained with 4% uranyl acetate and Reinold's lead citrate. The contrasted sections were imaged under a Hitachi H-7650 transmission electron microscope at 80 kV equipped with a 16 megapixel EMCCD camera (XR111, Advanced Microscopy Technique, MA, USA) was used for viewing the sections (Cho et al., 2007; Katragadda et al., 2009).

### Mitochondrial function

Non-infarct regions were used for assaying mitochondrial enzymatic function (complexes I–IV and Citrate synthase), using established spectrophotometric techniques (Samokhvalov et al., 2013). Heart powders were homogenized in ice-cold homogenization buffer (0.121 g of Tris, 0.15 g of KCl, and 0.038 g of EGTA in 50 ml of distilled water, pH 7.4, 0.854 g of sucrose /10 ml of the buffer was added at the day of the experiment), centrifuged at 600 g for 10 min at 4°C and supernatant was collected. Protein was then assayed using a Bradford reagent.

ATP content was assessed in non-infarct regions using a colorimetrically based ATP assay Kit (ab83355, Abcam Inc, Toronto, ON, Canada). Heart powders were homogenized and centrifuged at 15000 × g for 2 min and the resultant supernatant was assessed for ATP content. Standard curve for ATP and reaction mixture were prepared according the kit manual in a 96-well-plate and optical density (OD) was measured at 570 nm.

### Mitochondrial respiration

Mitochondria were freshly isolated from hearts according to established protocols (Shrestha et al., 2014). Briefly, heart homogenate was first centrifuged at 700 × g for 10 min followed by centrifuging the supernatant at 10,000 × g for 10 min, then the pellet was resuspended and washed using isolation buffer at 10,000 × g for 10 min. Mitochondrial oxygen consumption was measured in isolated mitochondria (50 ug of mitochondrial protein) added to a chamber connected to OXYGRAPH PLUS system (Hansatech Instruments Ltd, Norfolk, England). Respiration rates were measured at 30°C in 2 ml of respiration buffer. Basal respiration was recorded after the addition of 5 mM malate and 10 mM glutamate as substrates for basal oxidative respiration. ADP-stimulated respiration was initiated by addition of 0.5 mM ADP then recorded. The efficiency of coupled oxidative phosphorylation was calculated as the ratio between basal and ADP-stimulated respiration rates (Kuznetsov et al., 2008).

### Immunoblot analysis

Non-infarct regions of the LV were flash frozen using liquid nitrogen and crushed with a mortar and pestle on dry ice to be kept at −80°C. The heart powder was then homogenized in ice-cold homogenization buffer. Protein was resolved on SDS-polyacrylamide gels, transferred to nitrocellulose membranes and immunoblotted as previously described (Samokhvalov et al., 2013). Immunoblots were prepared using cytosolic (100 μg protein) or mitochondrial (25 μg protein) fractions and probed with antibodies to sEH (sc22344, Santa Cruz Biotechnology), SDH-A (ab5839s), CS (ab129095) (Abcam, Burlingame, CA, USA), COX IV (cs11967), and GAPDH (cs51745) (Cell signaling Technology, Inc., New England Biolabs, Ltd., Whitby, ON, Canada). Relative band intensities were assessed by densitometry using Image J (NIH, USA). Protein expression in vehicle treated controls were taken as 100% and compared with treated group.

### Measurement of glucose oxidation and fatty acid oxidation

Hearts from both sham-operated and post-MI mice were isolated and perfused in the working mode, as described (Rouslin, 1983; Larsen et al., 2012). Isolated working hearts were perfused at a left atrial preload of 11.5 mmHg and an aortic afterload of 50 mmHg with perfusate contained 5 mM [U-^14^C] glucose, 1.2 mM [9,10-^3^H]palmitate, and 3% albumin. The palmitate was prebound to 3% fatty acid free bovine serum albumin. First, hearts were subjected to an aerobic perfusion without insulin for first 30 min, then 100 μU/ml insulin was added to some hearts to investigate the response to insulin. In some perfusions, hearts were subjected to aerobic perfusion in the absence of insulin for the entire 60-min period. Rates of glucose oxidation and palmitate oxidation were determined by quantitative collection of ^14^CO_2_ and ^3^H_2_O from [U-^14^C] glucose and [9,10-^3^H] palmitate, respectively. At the end of the perfusion, hearts were frozen by liquid N_2_ and stored at −80°C until used for subsequent biochemical analyses (Barr and Lopaschuk, 1997; Belke et al., 1999). PI3-Kinase activity was assessed in lysates isolated from non-infarct regions of LV following LAD surgery using an ELISA based (5 μg protein) assay (Cat # K-1000s, Echelon Biosciences, Inc., UT, USA).

### Statistical analysis

Values expressed as mean ± standard error of mean (SEM). Statistical significance was determined by one-way ANOVA with Bonferroni *post-hoc* test was performed to assess differences between groups. Values were considered significant if *p* < 0.05.

## Results

### sEH inhibition improves cardiac function following myocardial infarction

**Figure 1:**
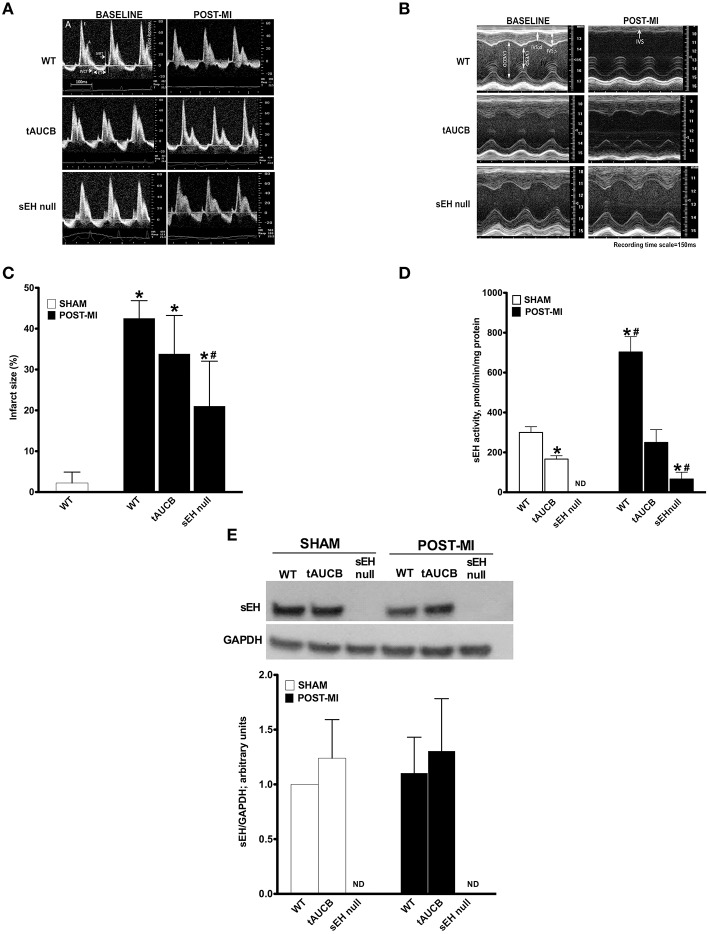
**Effect of sEH suppression on preserving ventricular dimensions**. Cardiac function of WT, tAUCB treated, and sEH null mice were assessed 1 day before LAD ligation (Baseline) and 7 days after LAD ligation (Post-MI). **(A)** Representative mitral Doppler spectrum showing E wave (early ventricular filling wave); A wave (a late filling wave caused by atrial contraction); IVCT, isovolumic contraction time; IVRT, isovolumic relaxation time; and ET, ejection time. **(B)** Representative M-mode images revealed a decrease in left ventricular dilation and dysfunction upon sEH inhibition. **(C)** Quantification of infarct size was assessed from images of transverse heart sections stained with TTC. **(D)** Non-infarct regions found in the left ventricle of hearts from sham and post-MI mice were assessed for sEH protein expression and **(E)** catalytic activity. Values represent mean ± SEM, *n* = 6–9, *p* < 0.05, ^*^significantly different from its baseline, ^#^significantly different from WT post-MI.

Baseline heart function in WT, tAUCB treated, and sEH null mice was similar among all groups (Figure 1A; Table 1). However, WT mice had significantly suppressed cardiac function following myocardial infarction compared to parallel tAUCB-treated and sEH null mice post-MI. At 7 days post-MI, WT mice showed LV dilation and systolic dysfunction as determined by increased LVESD and LV systolic volume, decreased EF, and FS. Inhibition of sEH either pharmacologically (tAUCB treated) or genetically (sEH null) attenuated the post-MI systolic dysfunction as shown by the significantly greater EF and FS. The post-MI increase in LVESD and LV systolic volume (LV Vol; s) was markedly diminished in sEH null and tAUCB-treated mice. Left atrial size (LA) was also increase in WT-MI mice accompanied with a decrease in mitral A-wave velocity, whereas sEH null and tAUCB-treated groups showed a significant attenuation in these parameters compared to WT-MI. We assessed the Doppler-derived myocardial performance index (TEI index), defined as the sum of isovolumic contraction time and isovolumic relaxation time divided by the ejection time index, and observed a marked increase in WT post-MI groups while inhibition of sEH prevented this increase. The attenuation of cardiac dysfunction by sEH inhibition was not accompanied by a significant reduction in infarct size expansion following tAUCB treatment, however infarct size was reduced in sEH null mice (Figure 1B).

**Table 1:** **Pre- and post-MI echocardiographic measurements of cardiac function in WT, tAUCB-treated, and sEH null mice**.

	**WT baseline**	**WT post-MI**	**tAUCB baseline**	**tAUCB post-MI**	**sEH null baseline**	**sEH null post-MI**
Heart rate (beats/min)	476.9 ± 17.4	430.3 ± 13.0	460.3 ± 19.0	426.8 ± 13.1	517.9 ± 19.8	493.1 ± 19.6
LVESD (mm)	2.31 ± 0.15	4.03 ± 0.3^*^	2.51 ± 0.22	3.37 ± 0.26	2.12 ± 0.15	2.87 ± 0.33
LVEDD (mm)	3.73 ± 0.12	4.81 ± 0.18^*^	3.66 ± 0.34	4.49 ± 0.14	3.59 ± 0.11	4.03 ± 0.16
LV Vol; d	56.9 ± 3.3	116.1 ± 7.4^*^	70.5 ± 5.6	87.3 ± 4.8^#^	54.7 ± 4.0	72.2 ± 7.6^#^
LV Vol; s	17.5 ± 2.5	83.7 ± 8.9^*^	26.9 ± 3.2	39.2 ± 3.1	15.6 ± 2.4	37.8 ± 8.5^#^
%EF	70.6 ± 2.6	28.2 ± 5.3^*^	62.9 ± 2.4	55.4 ± 1.8^#^	72.3 ± 3.2	49.4 ± 6.9^#^
%FS	39.7 ± 2	13.5 ± 2.7^*^	33.9 ± 1.8	28.7 ± 1.2^#^	41.3 ± 2.9	26.6 ± 4.3^#^
LV Mass	72.9 ± 3.6	77.4 ± 10.2	83.3 ± 6.3	80.0 ± 7.2	69.3 ± 3.9	86.0 ± 9.3
LA (mm)	1.6 ± 0.1	2.7 ± 0.2^*^	1.9 ± 0.2	1.9 ± 0.1^#^	2.1 ± 0.1	2.0 ± 0.2
Mitral E Vel (mm/s)	727.7 ± 35.4	638.6 ± 51.2	744.3 ± 30.3	628.8 ± 47.2	811.5 ± 25.4	677.7 ± 26.5
Mitral A Vel (mm/s)	445.3 ± 26.5	216.5 ± 53.1^*^	444.8 ± 39.8	480.7 ± 58.9^#^	485.7 ± 18.5	352.4 ± 27.2
IVRT (ms)	14.5 ± 1.8	18.5 ± 1.5	14.3 ± 2.2	15.6 ± 1.1	13.4 ± 0.5	14.7 ± 1.04
IVCT (ms)	10.7 ± 1	12.9 ± 2.2	8.6 ± 1.2	8.6 ± 1.0	7.1 ± 0.7	4.9 ± 0.8
ET (ms)	53.7 ± 1.9	49.8 ± 0.8	49.8 ± 1.4	52.7 ± 2.6	39.6 ± 3.3	44.6 ± 1.6
Tei index	0.47 ± 0.05	0.67 ± 0.06^*^	0.47 ± 0.06	0.41 ± 0.02	0.48 ± 0.03	0.42 ± 0.03

*Values represent mean ± SEM, n = 9, p < 0.05*,

* *significantly different from its baseline*,

# *significantly different from WT post-MI*.

sEH protein expression was not altered following tAUCB treatment in either sham or post-MI hearts, while no expression was detected in sEH null hearts (Figure 1C). Baseline catalytic activity was inhibited by tAUCB and absent in sEH null hearts. MI injury triggered an increase in sEH catalytic activity in WT hearts, which was inhibited by tAUCB and deletion of sEH (Figures 1D,E). The background hydrolysis of the substrate was expected as the assay was designed for recombinant and affinity purified sEH. The substrate will yield a fluorescent product when it reacts with glutathione, protein sulfhydryls, glutathione S-transferase, esterases, and other hydrolytic enzymes but not other known mammalian epoxide hydrolases.

### sEH inhibition protects the mitochondria from ischemic damage

**Figure 2:**
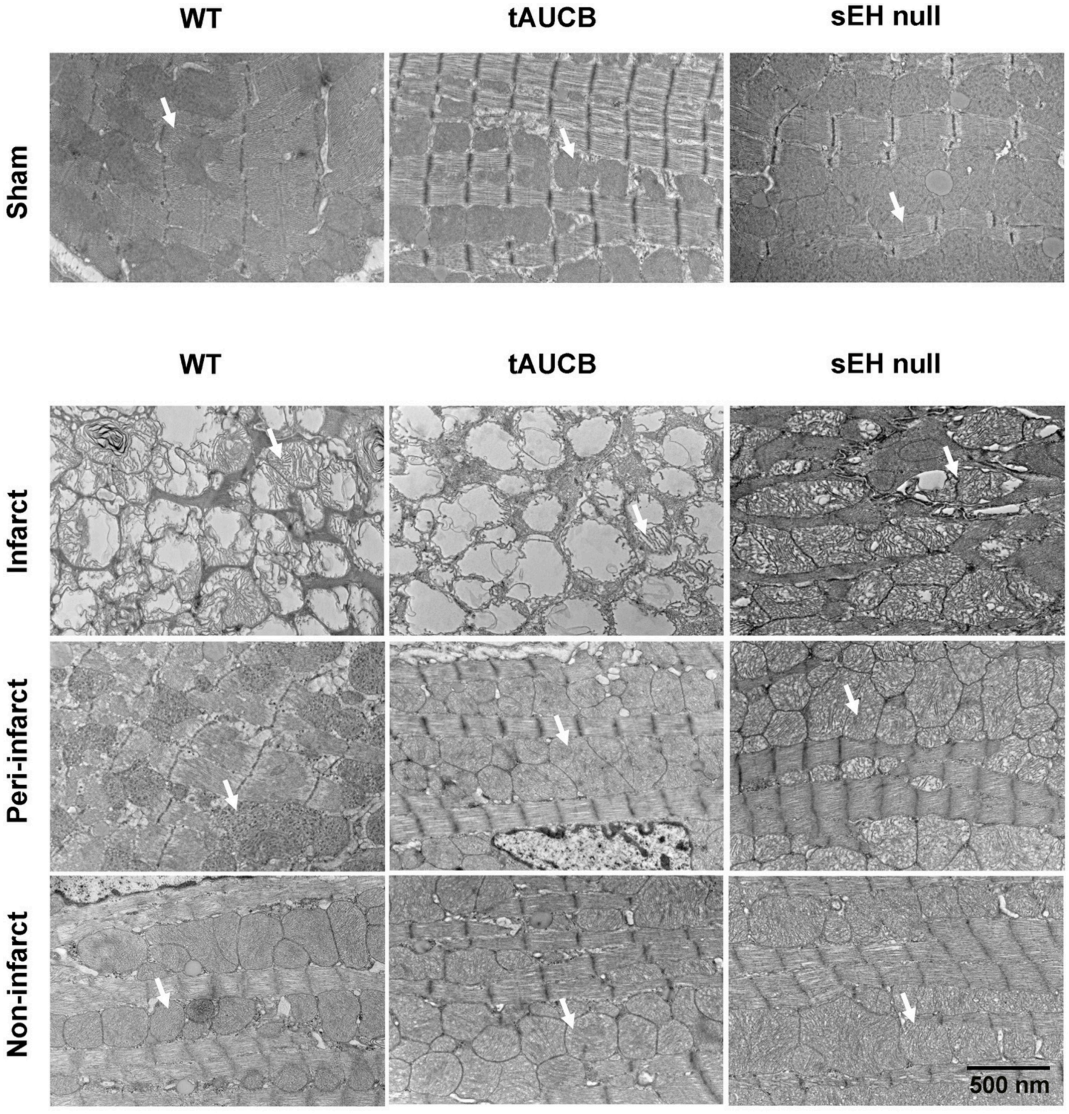
**Effect of sEH suppression on mitochondrial integrity**. Representative electron micrograph images of infarct, peri- and non-infarct regions found in the left ventricle of hearts. Arrows indicate individual mitochondrion (Magnification = 6000x).

To visualize the effect of ischemia on mitochondrial ultrastructure we assessed baseline and post-MI hearts using electron microscopy. Healthy and intact mitochondria were observed in the sham groups with no differences between WT, tAUCB, or sEH null mice. Seven days post-MI, we dissected the left ventricle into infarct, peri-, and non-infarct regions. EM images demonstrate that the mitochondrial ultrastructure of the infarct region in the sEH null group was significantly more preserved than the infarct regions of the WT and tAUCB treated groups. In the peri-infarct region, the mitochondrial content was more preserved than the infarct region in all the groups, moreover, mitochondrial damage was attenuated in tAUCB and sEH null mice compared to the WT group. The mitochondrial ultrastructure was not impacted in the non-infarct region of the three groups (Figure 2).

### sEH inhibition leads to preservation of mitochondrial efficiency in non-infarct region

**Figure 3:**
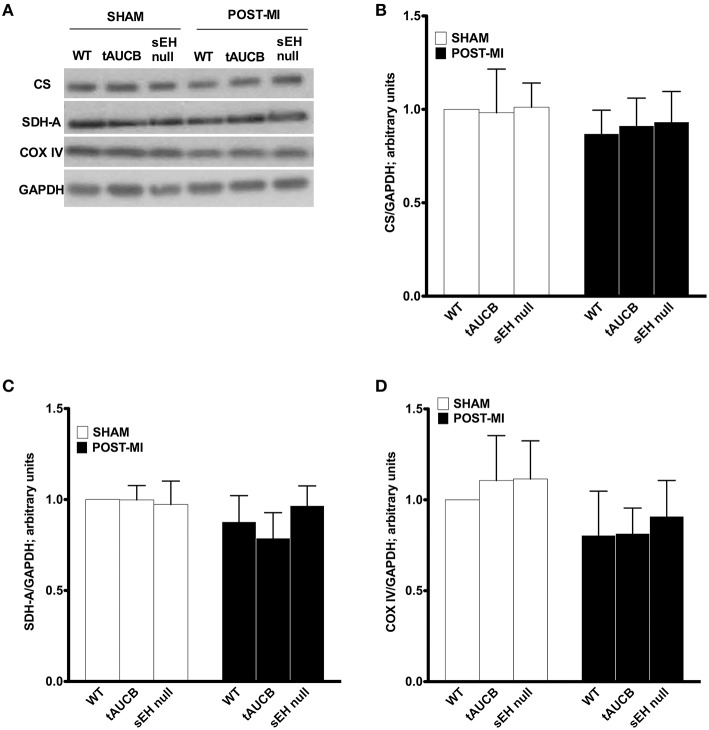
**Mitochondrial protein expression in the non-infarct region of the left ventricle**. Representative western blots **(A)** and quantification of **(B)** citrate synthase, **(C)** succinate dehydogenase (SDH-A), and **(D)** COX-IV protein expression observed in non-infarct regions of LV in sham operated and post-MI hearts. Values represent mean ± SEM, *n* = 4.

In order to maintain cardiac contractility and function, the heart needs a healthy pool of mitochondria to supply it with the energy required for contraction. We first quantified the abundance of key mitochondrial proteins in the non-infarct region where mitochondrial ultrastructure was preserved. Consistent with EM images, there was no significant difference in the protein content between any of the groups in either sham or post-MI for citrate synthase, succinate dehydrogenase or cytochrome C oxidase expression (Figure 3). These observations suggest the pool of mitochondrial protein found within the non-infarcted region remains the same in tAUCB and sEH null mice hearts relative to controls.

**Figure 4:**
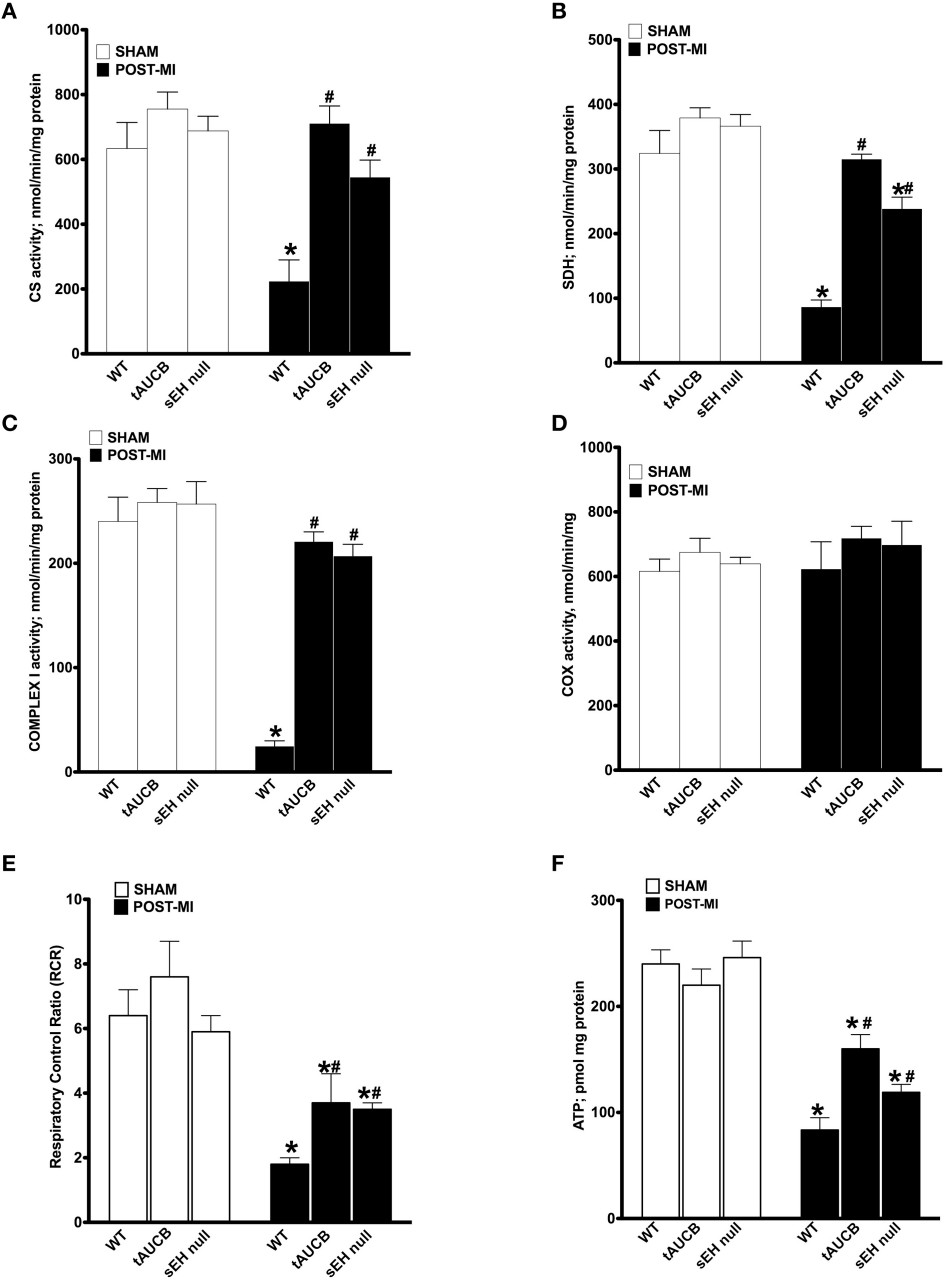
**sEH suppression preserves mitochondrial function following ischemic injury**. Activities of key mitochondrial enzymes were assessed spectrophotometrically in non-infarct regions of LV in sham operated and post-MI hearts. Activities of **(A)** citrate synthase, **(B)** succinate dehydrogenase, **(C)** complex I, and **(D)** cytochrome C oxidase were determined. **(E)** Respiration in isolated mitochondria was measured using Clark-electrode based chamber connected to oxygraph and rates are expressed as Respiratory Control Ratio (RCR). **(F)** ATP content was measured in the non-infarct region of the heart by a colorometric-based assay. Values represent mean ± SEM, *n* = 4, *p* < 0.05, ^*^significantly different from respective control sham, ^#^significantly different from WT post-MI.

Enzymatic activities of the mitochondrial respiratory chain were assessed in non-infarcted regions of the heart where no damage in the mitochondrial ultrastructure was observed. No significant differences were observed in the enzymatic activities between sham WT, tAUCB, or sEH null groups (Figure 4). However, there was a significant drop in citrate synthase (CS) activity in WT post-MI groups. This decrease was significantly attenuated in tAUCB treated and sEH null groups (Figure 4A). Similarly complexes I and II of the electron transport chain (ETC) showed a significant drop in their enzymatic functionality in WT post-MI groups, however, this was significantly attenuated in the tAUCB and sEH null mice (Figures 4B,C). Complex IV was preserved from ischemic dysfunction in both WT and treated groups post-MI (Figure 4D).

Considering sEH inhibition attenuated the loss of catalytic activity of key enzymes involved in ATP production, we next measured respiration in isolated mitochondria. To ensure respiration rates were not attributed to low substrate availability, malate, and glutamate were used to support basal respiration. WT post-MI groups showed a significant decrease in ADP-simulated respiration that was attenuated by inhibiting sEH with tAUCB or deletion in sEH null mice, which is reflected in the preserved respiratory control ratio (RCR). Enhancement in RCR was seen in the post-MI tAUCB and sEH null groups compared to WT mice (Figure 4E). Consistent with better mitochondrial respiration following inhibition or loss of sEH, ATP content in the non-infarct region of the LV was maintained in the tAUCB and the sEH null groups post-MI (Figure 4F).

### Inhibition of sEH and cardiac energy metabolism

**Figure 5:**
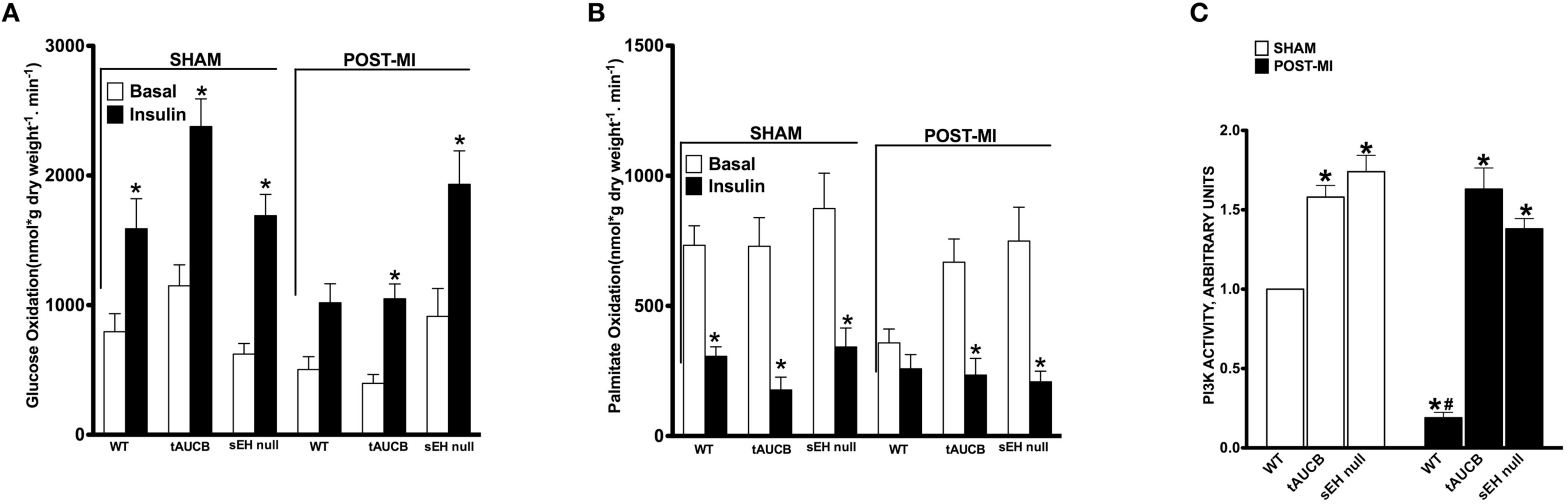
**The effect of sEH suppression on cardiac metabolism**. Rates of **(A)** glucose oxidation and **(B)** palmitate oxidation in the absence (basal) or presence of 100 μU/mL insulin from sham operated and post-MI hearts. Values represent mean ± SEM, *n* = 6, *p* < 0.05, ^*^significantly different from respective basal oxidation, **(C)** PI3K activity in non-infarct regions of LV from sham operated and post-MI hearts. Values represent mean ± SEM, *n* = 5, *p* < 0.05, ^*^significantly different from respective WT, ^#^significantly different from sham WT.

*Ex vivo* working hearts were used to investigate the effect of sEH inhibition on energy metabolism following MI. In the absence of insulin, the rates of glucose oxidation were similar between the experimental groups (Figure 5A). In response to insulin, all sham hearts showed a significant increase in glucose oxidation (fold increase WT 1.49, tAUCB 2.06, sEH null 2.21). However, only hearts from tAUCB treated or sEH null mice demonstrated a significant response to insulin following MI (Figure 5A) (fold increase WT 2.02, tAUCB 2.94, sEH null 2.11). Fatty acids are the primary energy substrate in the heart and fatty acid β-oxidation is closely and inversely coupled with glucose oxidation via the Randle cycle. While the rate of palmitate oxidation was unaltered in the absence of insulin, the rate significantly decreased in all sham hearts after adding insulin (Figure 5B) (fold decrease WT 2.4, tAUCB 4.11, sEH null 2.55). Damage from MI correlated with decreased basal palmitate oxidation in WT hearts compared to sham-operated mice but was not altered in hearts from tAUCB treated or sEH null mice. Moreover, palmitate oxidation was not altered after adding insulin in WT hearts post-MI (Figure 5B) (fold decrease WT 1.38, tAUCB 2.94, sEH null 2.6). Collectively, these data suggest that sEH inhibition preserved the cardiac response to insulin (i.e., insulin sensitivity) following MI. PI3K activity was significantly elevated in hearts from both tAUCB treated and sEH null mice (Figure 5C). A similar trend in increased Akt~P was observed in hearts but did not reach statistical significance (data not shown).

## Discussion

This study demonstrates that both pharmacological and genetic approaches to inactivate sEH preserves mitochondrial and cardiac function following ischemic injury. Moreover, inhibition of sEH maintained cardiac insulin sensitivity post-MI. Restoration of cardiac insulin sensitivity associated with inactivation of sEH suggests that the injured region of myocardium is undergoing robust structural and functional recovery. The ischemic insult did not affect mitochondrial structure and protein content in non-infarct regions, but did dramatically reduce mitochondrial function, which was prevented by inhibition of sEH. Accumulation of aberrant mitochondria triggers further dissemination of injury on intracellular structures and eventually, leads to cardiomyocyte death. Our results demonstrate a key role of protecting mitochondrial function in mediating protective effects associated with inactivation of sEH.

Myocardial infarction occurs when blood supply to the myocardium is interrupted as a result of coronary blockage or injury resulting in a state of energy starvation of the affected myocardial tissue. There are numerous detrimental consequences such as the development of mitochondrial crisis associated with defective cardiac metabolism eventually leading to heart failure (Lesnefsky et al., 2001). A large body of evidence has demonstrated a positive correlation between cardiac dysfunction in a failing heart attributable to decreased mitochondrial respiration rates. In this study we used the LAD occlusion model of MI (Virag and Lust, 2011). As expected, ligated WT mice showed a decrease in LV diastolic and systolic function and a marked reduction in cardiac contractility, which was preserved in tAUCB treated and sEH null mice. It has been well-documented that sEH inhibition increases the levels of endogenous EETs by suppressing their enzymatic degradation through sEH (Liu et al., 2009; Duflot et al., 2014). The ratio of EETs to DHETs in mice is elevated in the plasma of tAUCB treated (Liu et al., 2009) and sEH null mice (Seubert et al., 2006; Neckář et al., 2012). Previously published studies demonstrate genetic deletion of the sEH gene or direct pharmacological inhibition of sEH activity provides cardioprotection (Seubert et al., 2006; Xu et al., 2006; Monti et al., 2008; Li et al., 2009; Batchu et al., 2012a; Merabet et al., 2012; Shrestha et al., 2014). In a 3-week pressure overload murine model, Xu et al. demonstrated beneficial effects of the sEH inhibitors AEPU and AUDA in limiting cardiac hypertrophy (Xu et al., 2006). Similarly, inhibition of sEH has been shown to improve LV function and reduce remodeling in a murine model of chronic heart failure (Merabet et al., 2012). In the present study, coronary artery ligation produced significant infarcts in LV, which was attenuated by inhibiting sEH resulting improved LV function.

Our data indicated that sEH suppression preserves mitochondrial efficiency in the non-infarct region of the LV, maintaining a healthy pool of cardiac mitochondria that correlates with better contractility and functionality. Increased efficiency will supply the heart with sufficient amounts energy in the form of high-energy phosphates. Because as ATP production is primarily carried out by oxidative phosphorylation, damage to the ETC will lead to cardiac dysfunction. The failing heart becomes unable to produce sufficient amount of ATP to meet its contractile energy requirements (Neubauer, 2007). Our data show a decrease in ETC enzymatic function, RCR, and ATP content in WT post-MI hearts demonstrating mitochondrial dysfunction. The decreased mitochondrial function occurred in the non-infarct region as TEM images showed preserved mitochondrial ultrastructure. This suggests that the decline in mitochondrial function occurred prior to any remodeling or significant protein damage in WT mice. Interestingly, mitochondrial protein levels and ultrastructure in the non-infarct region were similar in all groups, but inhibition of sEH prevented the loss of mitochondrial function. Given citrate synthase and complex I activities serve as biomarkers for mitochondrial content our data suggests there is a preservation of the mitochondrial pool (Larsen et al., 2012). Ischemic injury in myocardium is known to decrease the activity of complex I of the ETC due to damage to an essential subunit in complex I (Rouslin, 1983). Complex I defect leads to electron leak and generation of ROS, which will eventually cause further damage to distal ETC complexes such as Complex III and IV (Lesnefsky et al., 2001).

In our model of a permanent coronary artery ligation we observed a marked activation of sEH catalytic activity, which resulted in increased conversion of EETs to the less active DHETs (Monti et al., 2008; Li et al., 2009; Liu et al., 2009; Chaudhary et al., 2010). Both our approaches, pharmacologically and genetic interruption of sEH, resulted in significant attenuation of enzymatic activity, supporting the notion that inhibition of sEH triggers a protective mechanism(s) preserving mitochondrial function within the heart. We have previously shown the beneficial effects of EETs and sEH inhibition involved improved mitochondrial function, which prevented the loss of mitochondrial membrane potential (ΔΨ_m_) and promoted cell survival (Katragadda et al., 2009; Batchu et al., 2011, 2012a; El-Sikhry et al., 2011). In a starvation model of cell injury EETs preserve ETC enzyme activities and mitochondrial protein content in HL-1 cells and neonatal cardiomyocytes (Samokhvalov et al., 2013). EETs can preserve mitochondria via activation of autophagy, which may shift death pathways toward survival, resulting in a healthier pool of mitochondria either through removal of damaged mitochondria or improvement of ETC (Samokhvalov et al., 2013). Importantly, we assessed mitochondrial function in non-infarct regions of the heart, and demonstrated no ultrastructure damage upon EM analysis. The observed enhanced ETC enzyme activities, oxygen consumption and ATP content would suggest inhibition of sEH preserved mitochondrial efficiency.

Preservation of post-MI insulin sensitivity following inhibition of sEH further supports the hypothesis of better mitochondrial efficiency and cardiac function. The normal heart can easily switch substrate utilization to meet energy requirements according to changes in hormonal levels or substrate availability (Lopaschuk et al., 2010). Insulin sensitivity represents the responsiveness of insulin receptors and downstream signaling in insulin-responsive tissues (Luria et al., 2011). Early stages of heart failure have been associated with significant reductions in insulin sensitivity and consequently, compromised glucose homeostasis (Ashrafian et al., 2007). Luria et al. demonstrated the role of sEH suppression in improving systemic insulin sensitivity and glucose homeostasis, where insulin sensitivity was preserved in both sEH null and tAUCB treated mice on a high fat diet (Luria et al., 2011; Iyer et al., 2012). They showed that sEH suppression stimulates insulin signaling in adipose tissue and liver due to activation of IRS-1 and PI3K (Luria et al., 2011). In the current study, we provide evidence that the cardiac response to insulin was blunted 7 days post-MI, which was preserved following sEH inhibition coincided with enhanced PI3K activity. Restoration in insulin sensitivity reflects preservation of mitochondrial function, which is supported by studies demonstrating the association between cardiac insulin resistance and decreased mitochondrial function (Boudina et al., 2009; König et al., 2012; Zhang et al., 2013). Cardiac dysfunction in both mouse models and human hearts of individuals with type 2 diabetes caused by systemic insulin resistance have significant mitochondrial defects including decreased mitochondrial respiration and ATP production (Boudina et al., 2009; Mansor et al., 2013). Similarly, deletion of the insulin receptor in mice to develop insulin resistance decreases cardiac contractility, which is associated with a reduction in ATP production and mitochondrial respiration (Boudina et al., 2009). A transverse aortic constriction model of heart failure produces cardiac insulin resistance leading to systolic dysfunction and exacerbation of contractile dysfunction (Zhang et al., 2013). Therefore, restoring the insulin sensitivity by sEH suppression can be associated with the better mitochondrial efficacy demonstrated in our results by the enhanced ATP production and RCR in a myocardial ischemia model.

The PI3K-Akt signaling pathway has a role in regulating insulin signaling, whereby phosphorylation of PI3K and Akt activate downstream mediators of the insulin cascade, including GLUT4 translocation, enhancing glucose metabolism (Okada et al., 1994; Tanti et al., 1996). Preserved insulin sensitivity, observed in sEH null and tAUCB-treated mice on a high fat diet, was associated with activation of IRS-1-PI3K-Akt axis in the liver and adipose tissue (Luria et al., 2011; Iyer et al., 2012). It has been well-established in previous studies that EET-mediated signaling involves activation of PI3K-Akt pathways limiting ischemia-reperfusion injury (Condorelli et al., 2002; Wang et al., 2005; Seubert et al., 2006; Batchu et al., 2012b). *In vitro* activation of the PI3K-Akt pathway was observed in EET-treated BAECs (Wang et al., 2005). Isolated murine hearts exposed to IR also demonstrated EET-mediated activation of PI3K. These results were consistent in sEH null mice (Seubert et al., 2006), as well as, mice hearts perfused with EETs (Batchu et al., 2012b) or sEHi (BI00611953) (Batchu et al., 2012a). Inhibition of PI3K or Akt results in shutting down the insulin cascade and is considered a primary cause in the development of insulin resistance (Okada et al., 1994; Tanti et al., 1996). Thus, activation of cardiac metabolism from one side and suppression of loss of mitochondrial function post-MI from another collectively result in promoting repair of myocardial structures and function. The enhanced PI3K activity observed in the current study indicates this as a potential mechanism by which sEH inhibition exerts its action.

In summary, here we demonstrate that pharmacological inhibition or genetic deletion of sEH mediates cardioprotective events following myocardial infarction through maintenance of mitochondrial efficiency. Our results show attenuation of sEH prevents cardiac dysfunction following MI by preserving the mitochondrial pool in the surviving (non-infarct) myocardium. Furthermore, inhibiting sEH preserved insulin sensitivity in post-MI hearts reflecting more optimal functioning cardiac metabolism thereby indicating activation of physiological recovery from ischemic insult.

## Author contributions

All authors listed, have made substantial, direct and intellectual contribution to the work, and approved it for publication.

## Funding

This work was supported by an operating grant from the Canadian Institutes of Health Research (JS MOP115037). KJ and AT are supported by graduate studentship awards from Alberta Innovates Health Solutions (AIHS, 201504). Partial support was provided by NIEHS RO1 ES002710 (BH).

### Conflict of interest statement

The authors declare that the research was conducted in the absence of any commercial or financial relationships that could be construed as a potential conflict of interest.
